# The relationship between body morphology, handgrip strength, and bone mineral density in Chinese college students: the mediating role of skeletal muscle

**DOI:** 10.7717/peerj.21735

**Published:** 2026-09-28

**Authors:** Zhilei Zhang, Xiujuan Liu

**Affiliations:** 1 School of Physical Education and Health, Heze University, Heze, China; 2 School of Political Science and Law, Heze University, Heze, China

**Keywords:** College students, Body morphology, Handgrip strength, Bone mineral density, Skeletal muscle

## Abstract

**Background:**

Adolescence is a critical period for bone mineral density (BMD) accumulation. However, the relationship between body morphology, handgrip strength (HGS), and BMD, as well as the mediating mechanisms involved, remains unclear among Chinese college students.

**Objective:**

To conduct an in-depth analysis of the mediating effect of skeletal muscle (the measured variable is skeletal muscle mass) on the relationship between body morphology, HGS, and BMD in Chinese college students, and to reveal the underlying mechanisms influencing human BMD.

**Methods:**

A random cluster sampling method was used to select 104 Chinese college students from a regular institution of higher education as cross-sectional study participants. Pearson correlation analysis and structural equation modeling were employed for data processing to construct a relationship model among height, weight, HGS, skeletal muscle, and BMD of the distal radius and ulna in the left forearm.

**Results:**

The Pearson correlation coefficients between height, weight, HGS, distal radius and ulna BMD, and skeletal muscle were 0.769, 0.795, 0.872, and 0.498, respectively (*p* < 0.05). After controlling for sex, skeletal muscle played a significant mediating role in the relationships between weight, HGS, and distal radius and ulna BMD among Chinese college students (*p* < 0.05), with mediating effect values of 0.293, and 0.238, respectively. Sex directly predicted skeletal muscle (*p* < 0.001) with effect values of 0.229, but not BMD (*p* = 0.056).

**Conclusion:**

Skeletal muscle plays a significant mediating role in the relationships between weight, HGS, and BMD in Chinese college students. Height does not indirectly influence BMD through skeletal muscle after controlling for sex. Increasing skeletal muscle mass remains an effective approach for enhancing BMD, particularly by maintaining adequate weight and grip strength.

## Introduction

Bone mineral density (BMD) is a key indicator of skeletal strength and bone health. College students are in a critical window for BMD accumulation, during which peak bone mass is attained (Zhang, Yang & Xue, 2000). Peak bone mass is the strongest predictor of future fracture risk, and its attainment is influenced by genetics, nutrition, and physical activity (Gordon et al., 2016). However, many college students maintain unhealthy lifestyles, prolonged sitting for academic reasons, poor dietary habits, and low physical activity levels (Han, Kim & Kim, 2021). These behaviors may negatively affect BMD not only during young adulthood but also in the long term. If these issues are not addressed, insufficient peak bone mass achieved by the end of young adulthood cannot be fully compensated for later in life, leading to an increased risk of osteopenia, osteoporosis, and fragility fractures in middle and old age (Chinese Society of Osteoporosis and Bone Mineral Research, 2019; Cheng & Lan, 2024).

Moreover, unhealthy behaviors often persist from childhood through adolescence, gradually leading to cumulative bone deficits. Therefore, understanding the mechanisms linking body morphology, muscle function, and BMD in college students is essential for designing early interventions that promote lifelong skeletal health.

Studies have indicated that BMD is significantly correlated with body morphology parameters such as height and weight, as well as body composition indicators like lean mass (Zhang et al., 2016); BMD, height, and weight are interrelated (Liu, 2002). Although most studies focused on middle-aged and elderly populations, overall BMD shows a significant positive correlation with physical indicators such as height, weight, and muscle mass (Yuan et al., 2022a). Height and weight are important factors influencing BMD. Taller individuals often possess a larger skeletal surface area, which may contribute to higher BMD. Moreover, height is a critical covariate when interpreting BMD Z-scores in growing populations, as it directly influences bone size and mineral content (Zemel et al., 2010). An increase in weight leads to a corresponding increase in BMD, as the mechanical pressure exerted by body weight on the skeleton stimulates an increase in BMD, thereby enhancing the bone's supportive capacity (Guo et al., 2016). An appropriate amount of weight gain may be beneficial for increasing BMD and play a positive role in preventing osteoporosis. Abdominal obesity shows a clear association with decreased BMD, with differences observed across various age and sex groups (Galvis et al., 2026). This suggests that the relationship between height, weight, and BMD may be influenced by multiple factors. As fundamental anthropometric measures, height and weight are closely related to an individual’s skeletal muscle mass and strength (Hou et al., 2021). The positive influence of height and weight on skeletal muscle is primarily manifested by providing the physical space and physiological demands necessary for muscle growth. Based on the above analysis, the following hypotheses are proposed: H1a: There is a close association between height and BMD; H2a: There is a close association between weight and BMD; H1b: Height has a positive effect on skeletal muscle; H2b: Weight has a positive effect on skeletal muscle.

Skeletal muscle mass is significantly correlated with handgrip strength (Das & Dutta, 2015; Safer et al., 2013); Genetic research shows certain genes are associated with low bone mineral density and low muscle mass simultaneously; cytokines affecting both muscle and bone cells have also been found in cell biology studies, and their altered levels directly cause declines in muscle mass and bone density (Li & Li, 2026). Skeletal muscle and bone mineral density are associated in adults (Kang, Kim & Do, 2022). Numerous studies have confirmed a positive correlation between skeletal muscle mass and BMD in adolescents and young adults (Cheng et al., 2024; Wang et al., 2023; Miyagi et al., 2024). A follow-up study comparing kidney transplant recipients with healthy controls aged 20–60 years showed that greater muscle mass was associated with larger increases in hip and spine BMD over a 2-year period (Ziolkowski et al., 2026). The positive correlation between skeletal muscle mass and BMD is an important physiological phenomenon, revealing a close link between muscle and bone health. However, research has found that individuals with higher muscle mass typically also have higher BMD, and increasing muscle mass may contribute to improving BMD (Xu et al., 2021; Hu et al., 2025). During strength training, muscle fibers are stimulated, leading to an increase in fiber size, *i.e*., muscle hypertrophy; thus, strength training positively influences skeletal muscle mass. The effect of handgrip strength (HGS) on BMD may be mediated through skeletal muscle. Therefore, the following hypotheses are proposed: H3: HGS is positively associated with skeletal muscle; H4: Skeletal muscle has a positive effect on BMD.

In summary, connections exist between human body morphology indicators (height, weight), physical function indicators (HGS), body composition indicators (skeletal muscle mass), and BMD. While substantial evidence for these connections comes from studies on middle-aged and elderly populations and individuals with osteoporosis, research focusing on college students remains relatively scarce. In particular, few studies have comprehensively examined the pathway with skeletal muscle serving as a mediating variable. Therefore, the aim of this study was to investigate the mediating role of skeletal muscle mass in the relationships between body morphology (height, weight), handgrip strength, and BMD in Chinese college students. Given that young adulthood is a crucial period for bone mass acquisition, which has a lasting impact on the attainment of peak bone mass, this study measured body morphology indicators (height and weight), a functional indicator (right HGS), a body composition indicator (skeletal muscle mass), and left forearm BMD in college students. The aim was to investigate the relationships among these indicators and propose a theoretical model. Given the well-documented sex differences in anthropometric measures, skeletal muscle mass, and BMD, we included sex as a covariate in the structural equation model to adjust for its potential confounding effect (Fig. 1). This approach seeks to explore the mechanisms influencing BMD in college students and provide further evidence for research on adolescent bone health.

**Figure 1:**
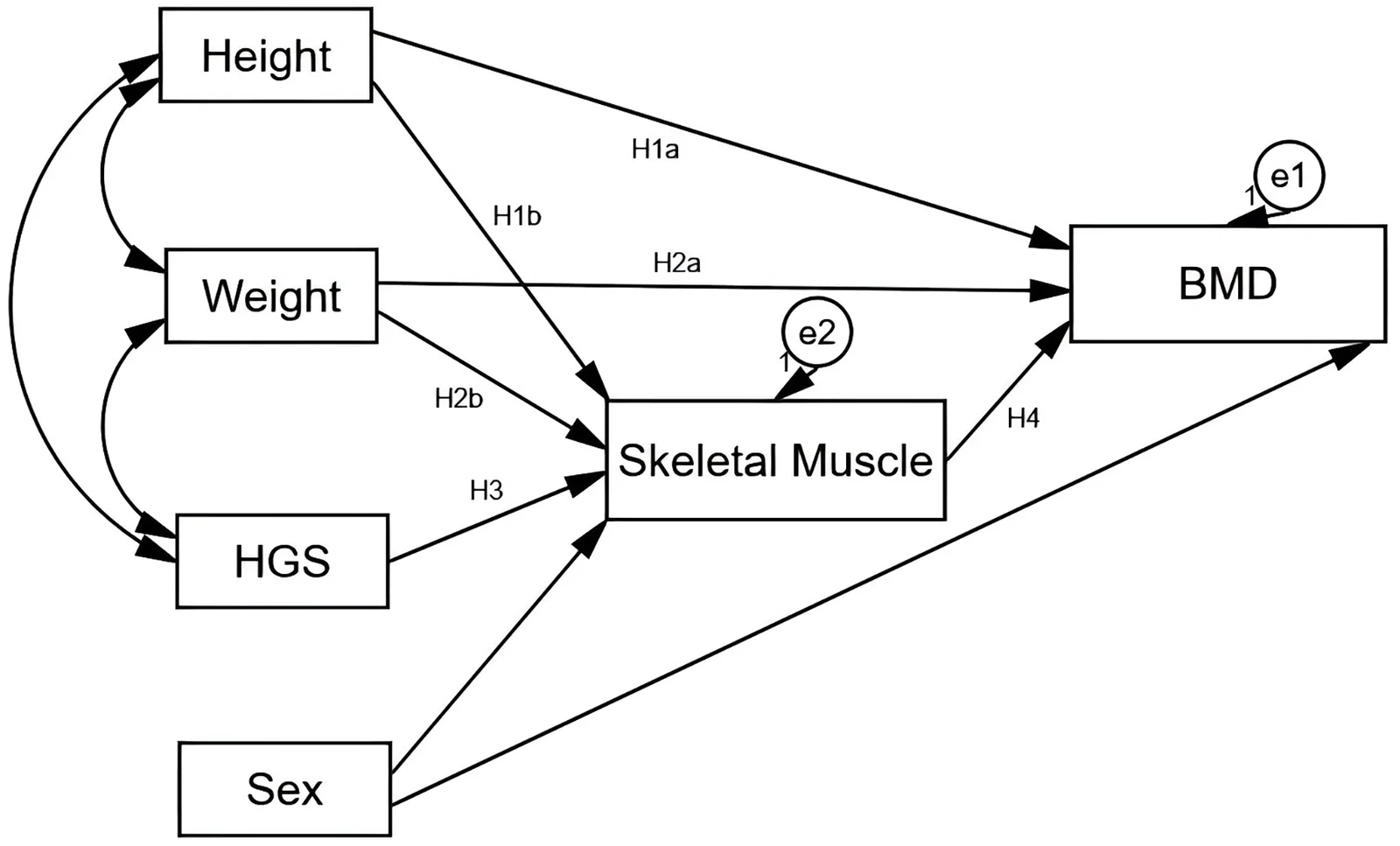
Theoretical model. Rectangles represent observed variables. Single-headed arrows denote hypothesized directional relationships. Height, weight, and HGS are proposed to affect BMD directly and indirectly *via* skeletal muscle mass, with sex included as a covariate.

## Materials and Methods

### Participants

This was a cross-sectional study. Sample size was estimated using G*Power 3.1 software. The recruited sample size also met the minimum recommended criteria for structural equation modeling. The study subjects were first-year students from a regular institution of higher education. Inclusion criteria were: first-year college students in good health and able to complete all tests. Exclusion criteria were: recent injury or illness preventing timely participation in the tests. One class each was randomly selected from two humanities majors, one science major, and one physical education major using cluster sampling, enrolling a total of 104 participants. This included 46 male students and 58 female students, with an age range of 18 to 22 years (mean age 19 years). This study was conducted in accordance with the Declaration of Helsinki. In accordance with the ethics committee’s approval of a waiver of written informed consent, all participants provided verbal informed consent before participating in the measurements. Participants recruitment was conducted from March 11 to 22, 2024. Measurement data collection took place on April 20–21, 2024, at the Sports and Health Laboratory of Heze University. All 104 participants completed all measurements, and complete case analysis was performed. The research received approval from the Academic Committee of Heze University (2022003), and the study is registered with the Chinese Clinical Trial Registry (Registration number: ChiCTR2000039041).

### Instruments and data collection

Height and weight were measured using standard Chinese National Physical Fitness instruments, including a stadiometer (Model: SGJ-II) and a weight scale (Model: TZJ-II). Skeletal muscle mass (kg) was measured using a Biospace body composition analyzer (Model: InBody770; Seoul, South Korea), which employs bioelectrical impedance analysis (BIA). This non-invasive method involves placing electrodes on the feet and hands; a low-frequency electrical current passes through the body, and its flow is influenced by body water content. The BIA device measures how the current signal is impeded by different tissue types (muscle has high conductivity, while fat slows the signal). After determining the resistance to the current flow through the body, the device uses equations based on body water values to calculate body composition data, including skeletal muscle mass (kg). Skeletal muscle mass was measured *via* InBody770 following a standardized 3-step protocol. First, participant preparation-fasting for ≥2 h, no vigorous exercise for 12 h, bladder emptying, no alcohol/caffeine for 24 h; second, electrode placement and posture–standing barefoot on foot electrodes, holding hand electrodes with arms slightly abducted (15–30°), legs slightly apart; third, multi-frequency acquisition—five frequencies (1 kHz to 1 MHz) applied to right/left segments, performed in a temperature-controlled room (22–24 °C), with automatic quality control. Bone mineral density was measured using an OsteoSys dual-energy X-ray absorptiometry device (Model: EXA-3000; Seoul, South Korea). BMD was measured at the distal radius and ulna of the left forearm. Handgrip strength was measured using a calibrated digital hand dynamometer (Model: WLJ-II). Participants stood upright with arms hanging naturally and elbows slightly flexed. Each participant performed two maximal voluntary contractions with the dominant hand (all participants reported being right-handed), lasting 3 s per trial, with a 30-s rest interval between trials. The maximum value (in kilograms) was used for statistical analysis.

All measurements were performed by the same trained technician in a professional laboratory setting.

### Statistical analysis

SPSS 23.0 (IBM, Armonk, NY, USA) was used for descriptive statistics and correlation analysis. SPSS 23.0 and Analysis of Moment Structures (AMOS) 24.0 (IBM, Armonk, NY USA) were used to construct the mediation model. The Bootstrap method (1,000 resamples) was used to test mediation effects, with 95% confidence intervals calculated. Statistical data are presented as mean ± standard deviation (M ± SD). A *p* < 0.05 was considered statistically significant. In the structural equation model, sex was included as a covariate to control for its potential confounding effects, and all analytical models were adjusted for sex. To minimize bias, we took the following steps: (1) trained staff conducted all measurements using calibrated, standardized instruments in a consistent laboratory setting; (2) random cluster sampling and strict eligibility criteria reduced selection bias; (3) structural equation modeling with bootstrap mediation testing accounted for potential confounding; and (4) data were double-entered and verified to prevent entry errors. We followed the STROBE guidelines for cross-sectional studies.

## Results

### Descriptive statistics and correlation analysis

The mean values of height, weight, HGS, skeletal muscle, and BMD among the surveyed college students, as well as the Pearson correlation coefficients, are presented in Table 1. HGS was strongly positively correlated with skeletal muscle mass (*r* = 0.872, *p* < 0.01), supporting a close link between muscle strength and mass in this young sample.

**Table 1:** Descriptive statistics and correlation coefficients for variables (*N* = 104, *M ±* SD).

Variable	*M ±* SD	Height	Weight	HGS	Skeletal muscle	BMD
Height (m)	1.680 ± 0.080	1				
Weight (kg)	62.713 ± 10.790	0.574**	1			
HGS (kg)	35.841 ± 11.318	0.731**	0.567**	1		
Skeletal muscle (kg)	25.266 ± 5.791	0.769**	0.795**	0.872**	1	
BMD (g/cm^2^)	0.453 ± 0.070	0.263**	0.307**	0.492**	0.498**	1

**Note:**

** *p* < 0.01.

### Structural equation model testing

Based on the research hypotheses and the proposed theoretical model, AMOS 24.0 was used to construct the structural equation model for validation. The maximum likelihood method was employed with AMOS 24.0 software to estimate the parameters of the theoretical model and test its goodness of fit. After adjusting for sex as a covariate, all key fit indices were acceptable, indicating good model fit (*p* > 0.05; Table 2). As shown in Table 3, sex had a significant direct effect on skeletal muscle (skeletal muscle mass) (*p* < 0.001) but not on BMD (*p* = 0.056). Height did not significantly predict skeletal muscle (*p* = 0.053), but it exerted a significant direct effect on BMD (*p* = 0.004). Weight significantly predicted skeletal muscle mass (*p* < 0.001) but had a non-significant direct effect on BMD (*p* = 0.514). Handgrip strength significantly predicted skeletal muscle mass (*p* < 0.001). Skeletal muscle mass significantly predicted BMD (*p* = 0.006).

**Table 2:** Fit indices of the data and model.

Fit index	*χ*^2^/df	RMSEA	*p* value	CFI	TLI	GFI	AGFI
Reference standard	≤3	<0.08	>0.05	>0.90	>0.90	>0.90	>0.90
Test value	0.409	<0.001	0.522	1.000	1.000	0.999	0.972

**Note:**

*χ*^2^/df = Chi-square/degrees of freedom ratio, a measure of how well the model fits the data relative to the complexity of the model. RMSEA = Root Mean Square Error of Approximation, a measure of the average discrepancy between the observed data and the model-predicted data. CFI = Comparative Fit Index, a measure of how well the model fits the data compared to a baseline model that assumes no relationships among the variables. TLI = Tucker-Lewis Index (previously called Non-normed Fit Index), a measure of model fit that adjusts for model complexity. GFI = Goodness of Fit Index, a measure of the proportion of variance in the observed data that is accounted for by the model. AGFI = Adjusted Goodness of Fit Index, a GFI that adjusts for the number of degrees of freedom, penalizing models with more parameters.

**Table 3:** Hypothesis test results for direct paths.

Hypothesized path	Standardized path coefficient	Unstandardized path coefficient	S.E.	C.R.	*p* value	Hypothesis test result
Height → BMD	−0.376	−0.329	0.113	−2.901	0.004	Supported
Weight → BMD	−0.105	−0.001	0.001	−0.652	0.514	Not supported
Skeletal muscle → BMD	0.649	0.008	0.003	2.736	0.006	Supported
Height → skeletal muscle	0.087	6.310	3.268	1.931	0.053	Not supported
Weight → skeletal muscle	0.451	0.242	0.020	12.202	<0.001	Supported
HGS → skeletal muscle	0.366	0.187	0.029	6.382	<0.001	Supported
Sex → skeletal muscle	0.229	2.657	0.601	4.423	<0.001	Supported
Sex → BMD	0.292	0.041	0.021	1.909	0.056	Not supported

**Note:**

S.E. stands for standard error; C.R. stands for Z-value.

### Mediation effect test

The Bootstrap method was employed to test the mediating role of skeletal muscle in the relationships between height, weight, HGS, and BMD among college students. Resampling was performed 1,000 times to calculate the 95% confidence intervals for the mediation effects. The bootstrap mediation analysis revealed that the indirect effects of weight (standardized effect was 0.293) and handgrip strength (standardized effect was 0.238) on BMD *via* skeletal muscle were statistically significant (Table 4). The indirect effect of height was not significant (95% CI included zero), indicating that height does not affect BMD *via* skeletal muscle mass after controlling for sex. The mediation model is presented in Fig. 2.

**Table 4:** Mediation effect test results.

Path relationship	Standardized effect value	Unstandardized effect value	Bootstrap 1,000 times 95% CI			
			Bias-corrected		Percentile	
			Lower	Upper	Lower	Upper
Height → skeletal muscle → BMD	0.056	0.049	−0.001	0.172	−0.012	0.145
Weight → skeletal muscle → BMD	0.293	0.002	0.078	0.575	0.076	0.568
HGS → skeletal muscle → BMD	0.238	0.001	0.074	0.412	0.070	0.410

**Note:**

Bootstrap resampling = 1,000 times. CI = confidence interval. The indirect effect of height is not significant at α = 0.05 because its confidence interval contains zero.

**Figure 2:**
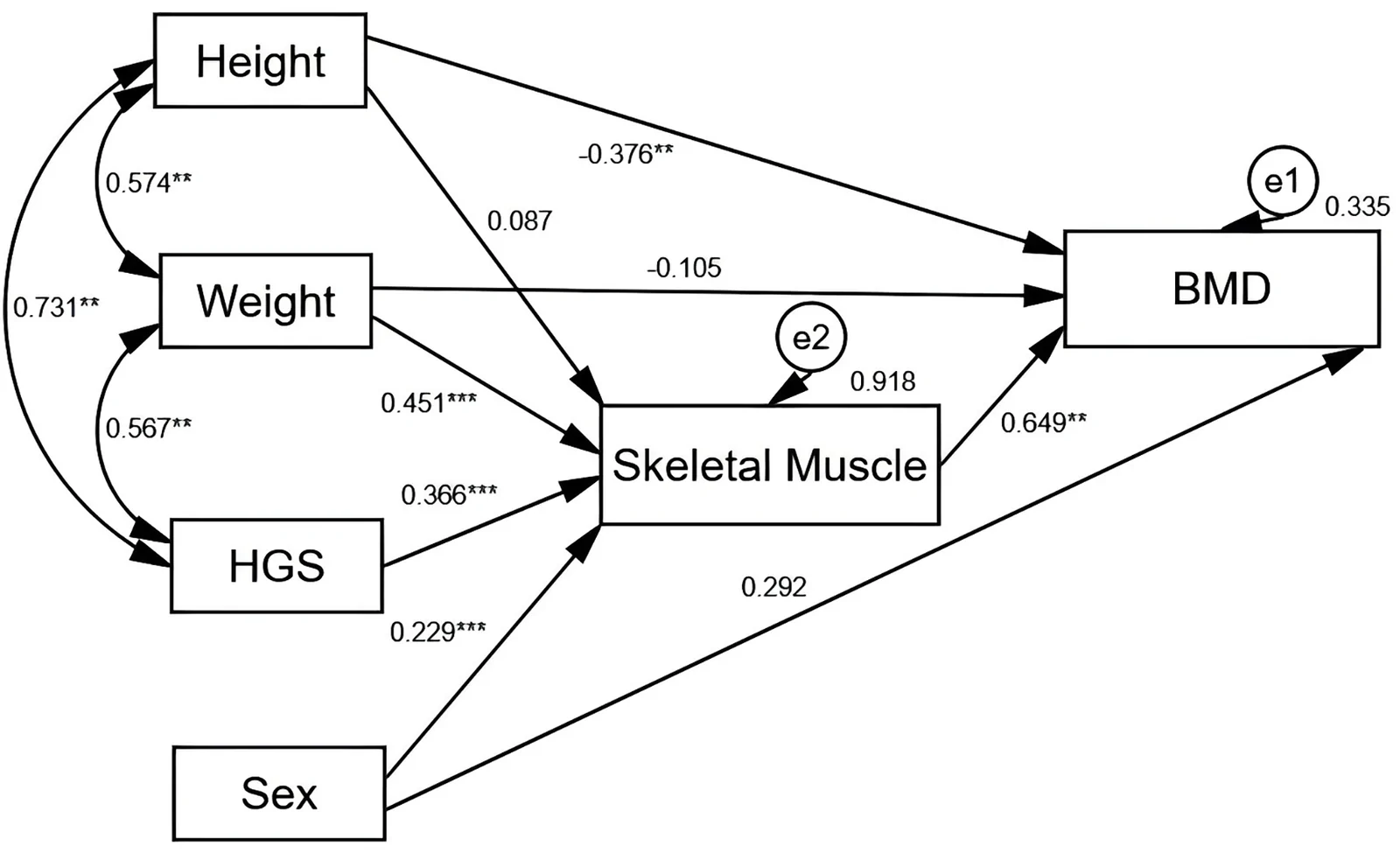
Mediation model. Structural equation model with standardized coefficients, testing the mediating role of skeletal muscle mass in the relationships between body morphology (height, weight), handgrip strength (HGS), and bone mineral density (BMD), with sex included as a covariate. Numbers on single-headed arrows are standardized regression weights (β). They indicate the direct effect of one variable on another, controlling for all other paths in the model. For example, the path from weight to skeletal muscle mass is β = 0.451, meaning one standard deviation increase in weight is associated with a 0.451 standard deviation increase in skeletal muscle mass. Numbers above the rectangles (here: 0.918 above Skeletal Muscle, 0.335 above BMD) are squared multiple correlations (R^2^). They represent the proportion of variance in the endogenous variable explained by all predictors pointing to it. For instance, 91.8% of the variance in skeletal muscle mass is explained by height, weight, HGS, and sex. e1 and e2 are residual (error) terms—the unexplained variance in BMD (e1) and skeletal muscle mass (e2). Abbreviations: HGS, handgrip strength; BMD, bone mineral density. **p < 0.01, ***p < 0.001.

## Discussion

The primary objective of this study was to test the mediating role of skeletal muscle mass in the relationships between body morphology (height, weight), handgrip strength, and BMD in Chinese college students. In this study of Chinese college students, after controlling for sex, we found that weight and handgrip strength positively influenced BMD through the mediating role of skeletal muscle mass, whereas height did not show a significant indirect effect. Sex directly predicted skeletal muscle mass but not BMD. These findings clarify how body morphology and physical function relate to bone health in young adults. All participants in this study were healthy college students without known bone metabolic diseases or recent injuries, which allows us to examine physiological relationships without the confounding effects of clinical conditions. Interestingly, the indirect effect of height on BMD *via* skeletal muscle mass was not significant after controlling for sex. This contrasts with our hypothesis and some previous studies that reported positive associations between height and BMD in unadjusted analyses. A possible explanation is that the height–BMD association is largely confounded by sex: males are generally taller and have greater muscle mass. After statistical adjustment for sex, residual height variance does not independently predict BMD *via* muscle mass. Alternatively, the relatively narrow height range in our college student sample may have limited the detection of a height effect. Future studies with more diverse height distributions are needed to clarify this issue. In this study, the Pearson correlation coefficients between height, weight, HGS, bone mineral density (BMD), and skeletal muscle among college students were 0.769, 0.795, 0.872, and 0.498, respectively (*p* < 0.05). Although our participants are healthy young adults, it is noteworthy that similar positive correlations between height, weight, and skeletal muscle mass have been observed in elderly patients with type 2 diabetes (Hou, 2019), suggesting that the muscle-bone relationship may be consistent across age groups. However, the mediating role of muscle mass in the weight-BMD relationship appears stronger in our healthy young cohort.

The contractile and weight-bearing actions of skeletal muscle may stimulate osteoblast activity through mechanical stress, thereby promoting increased BMD. This finding aligns with previous research results, as reported in several studies. For example, with advancing age, enhancing physical activity to increase skeletal muscle mass and strength, and maintaining limb muscle mass, may play a preventive role against bone loss or fracture occurrence (Lin, Asileng & Dong, 2022). The connection between bone and muscle deterioration in patients with musculoskeletal system decline is termed osteosarcopenia; for such patients, early engagement in muscle exercise would be meaningful (Schröder et al., 2023). Large-sample clinical studies have found that maintaining good muscle mass and fat mass in postmenopausal women contributes to improving overall BMD and total bone mineral content (Yuan et al., 2022b; Zhao et al., 2022). By the end of adolescence, various body systems are largely mature, and bone mass is in a phase of slow growth; however, under resistance training stimulation, BMD increases, indicating that appropriate loading stimulation can promote BMD enhancement (Li et al., 2019). Physical activity exerts an anabolic effect on bone tissue and can increase BMD in young people and adolescents (Courteix, 1998). Long-term exercise positively promotes bone mineral accumulation in both trabecular and cortical bone (Zheng, 2011). Research has shown that long-distance running may benefit regional BMD in university-aged males (Infantino, McCormack & Almstedt, 2021). Late adolescence is one of the critical periods for bone mass accumulation, lasting 2–5 years; appropriate physical training during this period helps maintain peak bone mass (Li, Wang & Yan, 2012). Adolescents need to engage in scientific and regular exercise, reduce body fat, and ensure dietary nutrition to improve overall BMD (Han, Kim & Kim, 2021).

This study has several strengths. First, it focuses on a poorly studied population healthy Chinese college students during a critical window for peak bone mass acquisition. Second, we applied structural equation modeling with bootstrap mediation and controlled for sex, providing more precise estimates than simple correlations. After controlling for sex, weight and handgrip strength influence BMD fully through skeletal muscle mass, whereas height does not. The non-significant indirect effect of height challenges the common assumption that taller individuals automatically have higher BMD *via* greater muscle mass. Instead, our results suggest that maintaining adequate weight and grip strength—proxy measures of muscle mass—is more important than height alone for bone health in young adults. Campus health programs should emphasize resistance training and protein-rich diets to increase skeletal muscle mass, rather than simply encouraging weight gain or focusing on height-related factors. Specific recommendations include integrating resistance exercise into physical education curricula, monitoring handgrip strength as a simple, non-invasive marker of muscle function, and educating students about the long-term benefits of peak bone mass attainment to prevent future osteoporosis. These actionable strategies can be implemented without expensive equipment and are suitable for large-scale campus interventions.

This study has several limitations. First, the participants were recruited from one university and one ethnic group in China. This may limit the generalizability of the findings to other populations or settings. Second, due to the limited sample size, sex-stratified analyses were not feasible. Instead, we included sex as a covariate in the structural equation model to statistically adjust for its potential confounding effect. While this approach is acceptable for detecting main effects, residual confounding by sex cannot be completely ruled out, and future studies with larger samples should perform sex-specific analyses. Third, we used BIA to measure skeletal muscle mass; dual-energy X-ray absorptiometry (DXA) is considered the gold standard for body composition assessment. Fourth, we did not collect detailed information on participants’ physical activity levels, which may influence both muscle mass and BMD. Future studies could address these limitations by using DXA, incorporating objective physical activity measurements, and including populations of different ages, ethnic backgrounds, and geographic regions.

## Conclusions

Height, weight, HGS, and BMD are positively correlated with skeletal muscle mass in college students. After controlling for sex, weight and HGS are positively associated with BMD through the mediating effect of skeletal muscle mass, whereas height does not indirectly influence BMD *via* skeletal muscle mass. These findings support practical strategies to improve BMD in young adults: regular resistance training to increase skeletal muscle mass helps maintain adequate body weight and grip strength, while mechanically stimulating bone formation. Resistance training should be incorporated into college physical education curricula to promote peak bone mass and long-term skeletal health. Handgrip strength can serve as a simple, non-invasive indicator of muscle function in campus screening.

## Supplemental Information

10.7717/peerj.21735/supp-1 Supplemental Information 1 Raw data.

## Additional Information and Declarations

### Data Availability

The following information was supplied regarding data availability:

The raw measurements are available in the Supplemental File.
